# Metabolic reprogramming involves in transition of activated/resting CD4^+^ memory T cells and prognosis of gastric cancer

**DOI:** 10.3389/fimmu.2023.1275461

**Published:** 2023-11-27

**Authors:** Yue Sun, Li Liu, Yuanyuan Fu, Yaoyao Liu, Xuan Gao, Xuefeng Xia, Dajian Zhu, Xiaping Wang, Xin Zhou

**Affiliations:** ^1^Department of Oncology, First Affiliated Hospital of Nanjing Medical University, Nanjing, China; ^2^Department of Gynecology, Shunde Women and Children’s Hospital (Maternity and Child Healthcare Hospital of Shunde Foshan), Guangdong Medical University, Foshan, Guangdong, China; ^3^Department of Pharmacy, First Affiliated Hospital of Nanjing Medical University, Nanjing, China; ^4^Department of Translational Medicine, Beijing GenePlus Genomics Institute, Beijing, China; ^5^State Key Laboratory of Microbial Resources, Institute of Microbiology, Chinese Academy of Sciences, Beijing, China; ^6^Department of Translational Medicine, Shenzhen GenePlus Clinical Laboratory, Shenzhen, China; ^7^Department of Gastroenterological Surgery, Shunde Women and Children’s Hospital (Maternity and Child Healthcare Hospital of Shunde Foshan), Guangdong Medical University, Foshan, China; ^8^Department of Pathology, The Second Affiliated Hospital of Nanjing Medical University, Nanjing, China; ^9^Oncology Center, The Affiliated Jiangsu Shengze Hospital of Nanjing Medical University, Suzhou, China

**Keywords:** all-trans retinoic acid, gastric cancer, metabolic reprogramming, tumor microenvironment, CD4+ memory T cells

## Abstract

**Background:**

Little is known on how metabolic reprogramming potentially prompts transition of activated and resting CD4^+^ memory T cells infiltration in tumor microenvironment of gastric cancer (GC). The study aimed to evaluate their interactions and develop a risk model for predicting prognosis in GC.

**Methods:**

Expression profiles were obtained from TCGA and GEO databases. An immunotherapeutic IMvigor210 cohort was also enrolled. CIBERSORT algorithm was used to evaluate the infiltration of immune cells. The ssGSEA method was performed to assess levels of 114 metabolism pathways. Prognosis and correlation analysis were conducted to identify metabolism pathways and genes correlated with activated CD4^+^ memory T cells ratio (AR) and prognosis. An AR-related metabolism gene (ARMG) risk model was constructed and validated in different cohorts. Flow cytometry was applied to validate the effect of all-trans retinoic acid (ATRA) on CD4^+^ memory T cells.

**Results:**

Since significantly inverse prognostic value and negative correlation of resting and activated CD4^+^ memory T cells, high AR level was associated with favorable overall survival (OS) in GC. Meanwhile, 15 metabolism pathways including retinoic acid metabolism pathway were significantly correlated with AR and prognosis. The ARMG risk model could classify GC patients with different outcomes, treatment responses, genomic and immune landscape. The prognostic value of the model was also confirmed in the additional validation, immunotherapy and pan-cancer cohorts. Functional analyses revealed that the ARMG model was positively correlated with pro-tumorigenic pathways. *In vitro* experiments showed that ATRA could inhibit levels of activated CD4^+^ memory T cells and AR.

**Conclusion:**

Our study showed that metabolic reprogramming including retinoic acid metabolism could contribute to transition of activated and resting CD4^+^ memory T cells, and affect prognosis of GC patients. The ARMG risk model could serve as a new tool for GC patients by accurately predicting prognosis and response to treatment.

## Introduction

Gastric cancer (GC) is a malignancy ranking among the world’s top five most common cancers, and is one of the three leading cancer-related deaths globally (1, 2). In recent years, various measures have been taken worldwide to reduce morbidity and mortality as part of public health policies (3). Great efforts have been made in early diagnosis and treatment of the disease with middle and late stages, resulting in declines for both parameters in several developed countries (4). However, the reduction in GC incidence remains insignificant in several countries including Latin America, Asia, and Eastern and Central Europe (5). The number of early-stage GC patients tends to increase and patients with advanced stage experience a poorer prognosis (6).

After years of intensive research, the direction of malignancy research has evolved from the simple study of tumor cells themselves to a complex ecological network that extends from within the tumor to the whole body, called the tumor microenvironment (TME) (7). TME refers to tumor cells consisting of extracellular matrix (ECM), stromal cells, and immune and inflammatory cells (8). The components of this ecological network interact and influence each other, as immune cells occupy a very important position (9). In previous studies exploring immune cells, some specific types such as CD8^+^ T cells, regulatory T cells (Treg cells) and macrophages were once the hot spots of research (10–12). However, recent research has shown that CD4^+^ T cells, particularly CD4^+^ memory T cells, are crucial for the immunotherapy-induced tumor regression (13).

Metabolic reprogramming, a frequent term for a collection of aberrant metabolism pathways seen in cancer cells with a high level of proliferation, stands as a sign of malignancy (14, 15). The interaction of metabolic reprogramming with TME and its role in GC have received increasing attention in recent years (16, 17). Reprogrammed energy metabolism, on the one hand, affects the course of GC and helps to create an immunological milieu that promotes tumors (18). On the other hand, aberrant signaling pathways or trophic competition in TME may result in phenotypic reprogramming of metabolic and functional changes in tumor-infiltrating immune cells, and then compromise the therapeutic effectiveness of cancer immunotherapy (19).

Metabolism is critical for the differentiation and effector functions of T cells, but aerobic glycolysis in cancer cells limits glucose consumption of T cells, thereby inhibiting their functions (20). Simultaneously, cellular metabolism (particularly lipid metabolism) is responsible for the development, survival, and effector activities of T cells, which also improves tumor immunotherapy (16, 21). It may become more important to explore how the TME of GC can be altered from the perspective of metabolic reprogramming to find new therapeutic targets as well as mechanisms of action, and subsequently identify new immune checkpoint modulators.

This research used bioinformatics models to assess CD4^+^ memory T cells in GC patients and investigated the connection between metabolic reprogramming and the activated CD4^+^ memory T cells ratio (AR). The study also delved into the roles and potential functions of the risk model developed from metabolism-related genes in GC and pan-cancer. Furthermore, we also explored the effect of retinoic acid metabolism pathway (one of the significant metabolism pathways) on the transition of AR in GC patients. The results will contribute to the development of precise therapeutic strategies for patients with GC.

## Materials and methods

### Datasets and case sources

The Cancer Genome Atlas (TCGA) database (https://portal.gdc.cancer.gov/) was used to retrieve RNA sequence, mutation, copy number variations (CNV), and clinical data. The Gene Expression Omnibus (GEO) (https://www.ncbi.nlm.nih.gov/geo/) database’s public microarray dataset and related clinical information for GC patients were located and downloaded. Age, gender, pathological TNM stage, and survival statistics were all included in the clinical data. Three GEO datasets (GSE84437, GSE57303 and GSE62254) and the TCGA-STAD dataset were selected and merged into one cohort for further analysis. As external validation, another GEO dataset (GSE15459) was used. The collection matrix documents and records tables for the microarray platform can be downloaded from the GEO website. Data have been preprocessed to exclude samples without complete clinical data.

### Estimation of immune cell type scores

The TCGA and GEO datasets were derived from multiple studies spanning different cell lines and platforms, with batch effects and noise. Therefore, the four datasets were co-normalized into one cohort using combat normalization in the “sva “R package. To measure the extent of immune cells in GC patients, we transferred standardized quality articulation information with standard comments to the CIBERSORT entry (https://cibersort.stanford.edu/). In the subsequent Kaplan-Meier (K-M) analysis, only samples with total CD4^+^ memory T cells (resting CD4^+^ memory T cells plus activated CD4^+^ memory T cells) > 0 were included. AR was defined as the activated CD4^+^ memory T cells ratio, with the formula: activated CD4^+^ memory T cells/total CD4^+^ memory T cells *100%.

### Identification of metabolism pathways affecting AR and construction of the predictive model

The 114 metabolism pathways and metabolism-related genes associated with these pathways were collected from the previous literature (22). We calculated the enrichment scores of metabolism gene sets by single-sample gene set enrichment analysis (ssGSEA) with the R package “GSVA” (23). By Cox regression and Pearson correlation test, pathways significantly correlated with resting CD4^+^ memory T cells, activated CD4^+^ memory T cells, AR, and prognostic correlation (P < 0.05) were screened. A venn diagram was used for determining prognostic and AR related intersection pathways. The AR-related metabolism gene (ARMG) prediction model was constructed by further downscaling the relevant genes through LASSO-Cox regression, and we generated ARMG risk score using the following algorithm: ARMG risk score = Σ Cox coefficient of gene Xi × scale expression value of gene Xi. Cytoscape was used to established protein-protein interaction (PPI) networks among ARMGs.

### Predictive validity of the ARMG model

Excluding cases with incomplete clinical data, the predictive validity of the ARMG risk score was validated in the training cohort. K-M curves were applied to compare overall survival (OS) differences according to the risk score, and PCA were used to verify group clarity. Besides, ROC curves compared ARMG risk score with AUC values for clinical variables. In all cohorts, we conducted univariate Cox regression (uniCox) and multivariate Cox regression (multiCox) analysis to ascertain if the ARMG risk score was an independent prognostic predictor. Then, based on numerous clinical characteristics, we conducted a classification study to determine whether the ARMG risk score maintained its capacity to predict outcomes across various subgroups. Specifically about OS at 1, 3, and 5 years, the nomogram was created to offer valuable clinical forecasts for GC patients, including the ARMG risk score and other clinicopathological traits. The established clinical dependability was then validated using a decision curve analysis (DCA). Additionally, we externally validated the OS in GSE15459.

### Immunotherapy and chemotherapy sensitivity prediction

The IMvigor210CoreBiologies package in the R program contains immunotherapy cohort data for urothelial bladder cancer (BLCA) is retrieved from the IMvigor210 database (http://research-pub.gene.com/IMvigor210Core Biologies/packageVersions/) (24), and was used to predict immunotherapy sensitivity for validation of ARMG risk score subgroups. Additionally, immunophenotype scores (IPS) (25) and tumor immune dysfunction and exclusion (TIDE) (26) were evaluated in GC patients to predict immunotherapy response. The half-maximal inhibitory concentration (IC50) from Cancer Genome Project (CGP) was calculated by the R package “pRRophetic” to assess response to chemotherapeutic agents. The sensitivity of chemotherapeutic agents among different risk groups was also analyzed in the Cellminer database (https://discover.nci.nih.gov/cellminer/home.do).

### Multi-omics analysis and functional analysis of ARMG risk score

The “maftools” package was used to construct the mutation annotation format (MAF) to compare the mutational profile of GC patients among various risk groups. The ARMG risk score, tumor mutation burden (TMB) scores, and the differences in OS between different subgroups were examined. In addition, the level of immune cell infiltration and immune checkpoints (ICP) in different subgroups were compared. We examined the correlation among ARMG model and cancer stem cell (CSC) scores, TME, N6-methyladenosine (m6A) related-, and cuproptosis related-genes. The enrichment of Gene Ontology (GO) and Kyoto Encyclopedia of Genes and Genomes (KEGG) pathways in both groups were evaluated with the ‘clusterProfiler’, ‘org.Hs.eg.db’, ‘enrichplot’ packages. The enriched biosynthetic pathways and processes with P < 0.05 and FDR q < 0.05 were considered statistically significant.

### Pan-cancer analysis of ARMG risk score

The pan-cancer study was performed based on the UCSC Xena (https://xena.ucsc.edu/) database’s genomic information for 33 different cancer types, including single nucleotide variation (SNV) and CNV data together with associated clinical information. We investigated the expression distribution of the prediction model in each cancer type. K-M curves were used to validate the predictive validity of the model in each cancer type in terms of OS, DFS, and PFS, and the correlation with clinical parameters. In addition, we validated the correlation of ARMG risk score with TMB, MSI, neoantigens, and immune microenvironment from a pan-cancer multi-omics perspective.

### Ethics statement and sample sources

Patients provided written informed consent for this study, which was authorized by ethics review committee in The First Affiliated Hospital of Nanjing Medical University (Ethics Approval No. 2022-SRFA-086). Six peripheral blood samples were collected from GC patients with advanced stages before initial treatment. These six patients were excluded from autoimmune system diseases and had no history of surgery, chemotherapy, radiotherapy or immunotherapy. Similarly, peripheral blood was collected as a control group from six healthy individuals who underwent a well-established routine examination to exclude malignancies, immune system and metabolic disorders. Peripheral venous blood drawn with EDTA anticoagulation before treatment was subjected to isolate mononuclear cells (PBMC), using the Ficoll-Hypaque gradient centrifugation method.

### Flow cytometry analysis

The cell suspensions were co-cultured with all-trans retinoic acid (ATRA, 10nM) or NC for 72 hours. Both groups were treated with the following particular antibodies for 30 minutes at room temperature: FITC anti-human CD3 (catalog no. 300305, Biolegend, USA), APC anti-human CD45RA (catalog no. 304111, Biolegend, USA), PE anti-human HLA-DR (catalog no. 307605, Biolegend, USA), PerCP/Cyanine5.5 anti-human CD4 (catalog no. 300529, Biolegend, USA). All samples were analyzed using a BD Biosciences Influx cell sorter. We counted CD3^+^CD4^+^CD45RA^-^HLA-DR^+^ cells to estimate levels of activated memory CD4^+^ T cells, and subtracted these values from total memory CD4^+^ T cells (CD3^+^CD4^+^CD45RA^-^) to quantify resting memory CD4^+^ T cells (27).

### Statistical analysis

The data are processed, analyzed, and presented using R language (version 4.1.2) and its pertinent packages. A two-sided P < 0.05 was considered significant. The mean and standard deviation (SD) are used to express all flow cytometry data. One-way analysis of variance (ANOVA) was used for the statistical analysis, and GraphPad Prism was used to process and analyze the data (version GraphPad Prism 9).

## Results

### Prognostic significance of CD4^+^ memory T cells in GC

**Figure 1:**
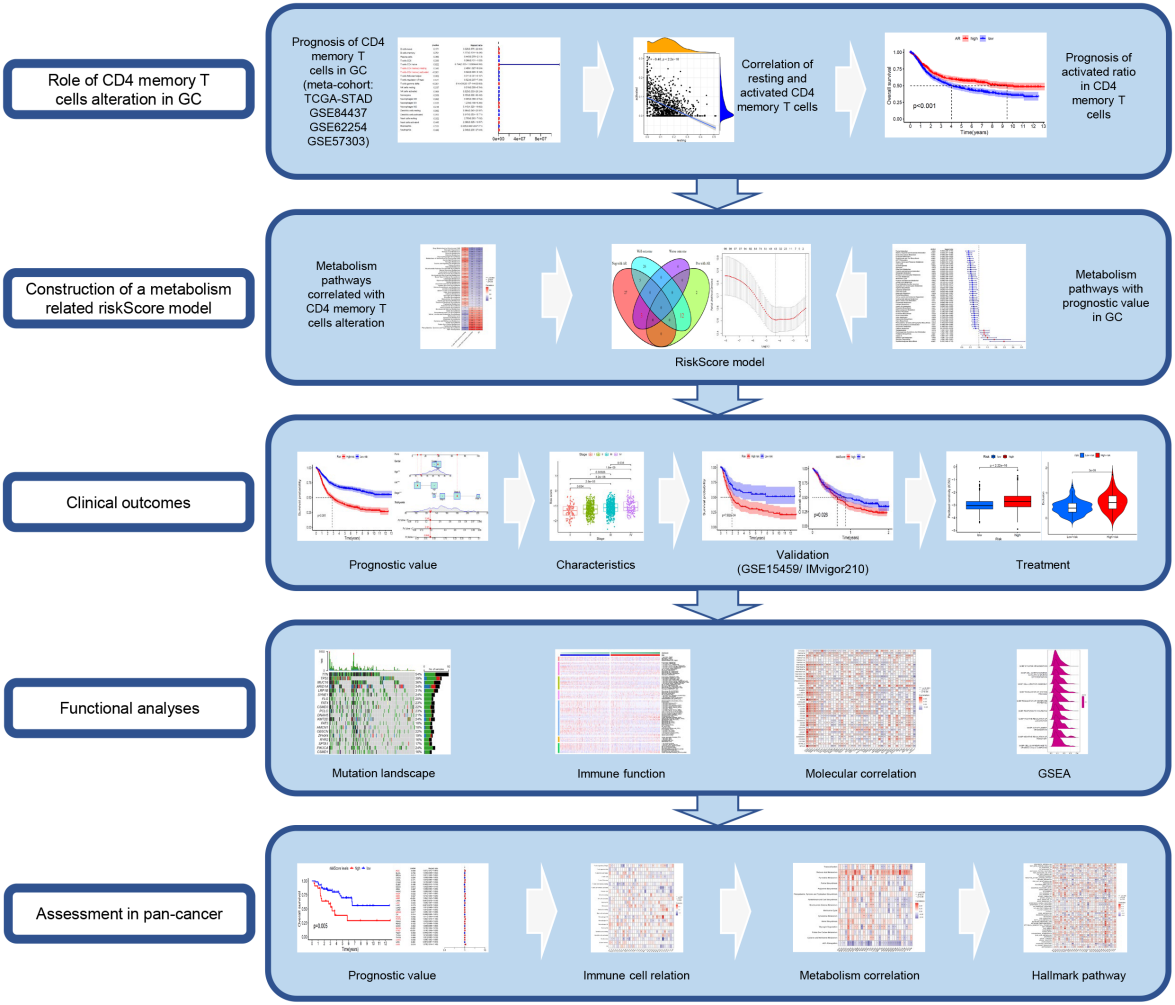
The workflow of the study.

**Figure 2:**
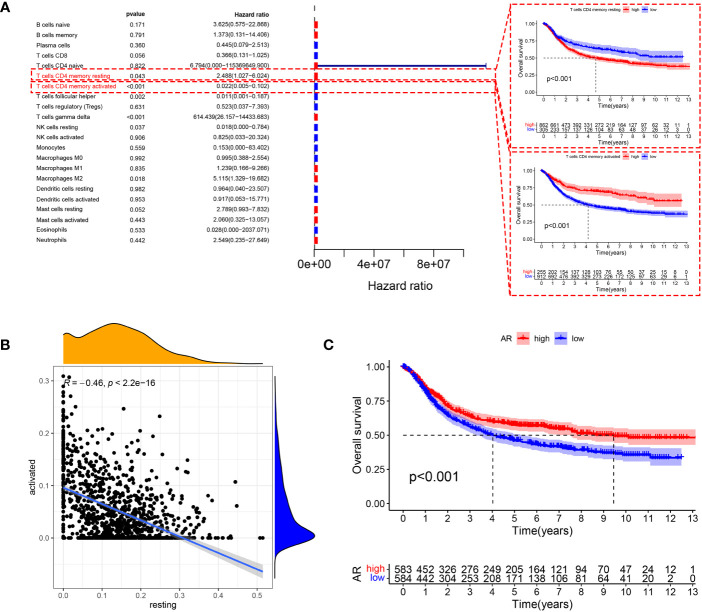
**(A)** Forest plot showing prognosis-related immune cells screened using the testing cohort and K-M curves showing prognostic differences in the expression of two types of CD4^+^ memory T cells. **(B)** Resting CD4^+^ memory T cells are negatively correlated with the expression of activated CD4^+^ memory T cells. **(C)** The level of AR also showed significant differences in prognosis, with patients with high AR having better OS.

The main workflow of this study is shown in **Figure 1**. Using the CIBERSORT algorithm, this study evaluated the infiltration levels of 22 immune cell types in GC samples and assessed their prognostic significance using the univariate Cox regression analysis (**Figure 2A**). Meanwhile, we found that resting CD4^+^ memory T cells and activated CD4^+^ memory T cells had opposite effects on OS. Specifically, lower levels of resting CD4^+^ memory T cells and higher levels of activated CD4^+^ memory T cells were associated with better prognosis (P < 0.05, **Figure 2A**). Therefore, we speculated that resting and activated CD4^+^ memory T cells were negatively correlated. The Pearson correlation curve (**Figure 2B**) validated the hypothesis, suggesting the possible transition of CD4^+^ memory T cells between the two states. Therefore, we further examined how AR affected prognosis. The result demonstrated that AR was significantly correlated with OS (P < 0.001, **Figure 2C**), and higher AR indicated better prognosis.

### Construction of ARMG risk model

**Figure 3:**
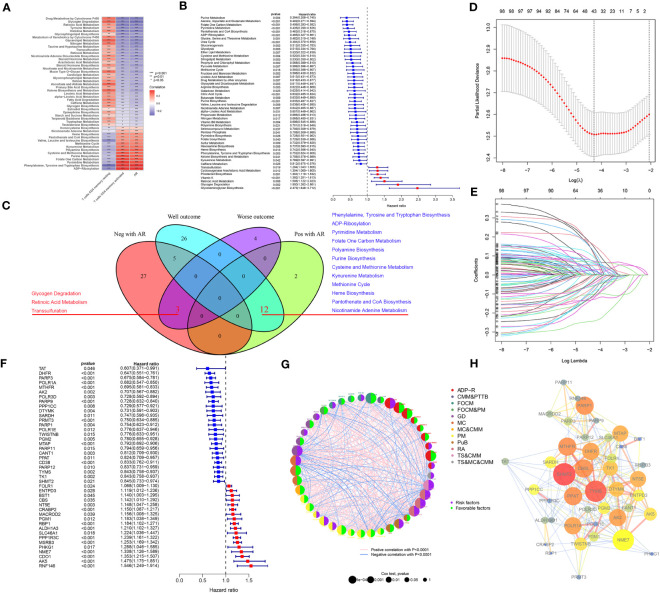
**(A)** Pearson correlation test screened 49 metabolism pathways associated with resting CD4^+^ memory T cells, activated CD4^+^ memory T cells and AR. **(B)** Screening of 50 metabolism pathways with prognostic relevance using testing cohort. **(C)** Venn diagrams showed that 3 metabolism pathways were negatively associated with AR and had a poor prognosis, and 12 metabolism pathways were positively associated with AR and had a good prognosis. **(D)** The change curve of penalty term. **(E)** The path change chart of the regression coefficient. **(F)** 43 metabolism-related genes were screened for use in constructing the model, and the forest plot shows the hazard ratio of the genes. **(G)** Correlations between 43 genes and the metabolism pathways that interact with each other. **(H)** PPI network of the 43 genes in the model, the higher the number of connected nodes, the deeper the color of the nodes.

To investigate the metabolism pathways involved in transition of resting and activated CD4^+^ memory T cells, we examined the correlation between CD4^+^ memory T cells and 114 metabolism pathways. A total of 49 metabolism pathways were simultaneously correlated with CD4^+^ memory T cells and AR, of which 14 were positively and 35 were negatively correlated with AR (**Figure 3A**). On the other hand, Cox regression analysis screened 50 metabolism pathways significantly associated with OS of GC (**Figure 3B**), of which 43 were associated with favorable outcomes and 7 with unfavorable OS. To study the metabolism pathways associated with both AR and prognosis, we intersected the four conditions that were positively or negatively associated with AR and prognosis. The Venn diagram showed that there were 12 pathways positively associated with both AR and favorable prognosis, and 3 pathways including retinoic acid metabolism pathway were negatively associated with AR and exhibited poor prognosis (**Figure 3C**).

Correlation analysis between AR and 303 genes derived from the 15 pathways was additionally performed to identify AR-related metabolism genes. A total of 216 ARMGs were subjected to lasso regression analysis to screen genes for construction of ARMG risk model. And 43 genes with 18 risk factors and 25 protective molecules were finally identified (**Figures 3D–F**). All the 43 ARMGs had significant interactions with at least one other gene (**Figure 3G**). A PPI network diagram of their interactions was presented in **Figure 3H**. In addition, these 43 genes had significant expression differences between normal and tumor samples in the TCGA-STAD dataset (**Figure S1A**) and showed varying levels of CNV gain and loss (**Figure S1B**). The chromosomal loci and the tumor mutation load of these genes were shown in **Figures S1C, D**.

### Validation of the predictive power of ARMG risk model

**Figure 4:**
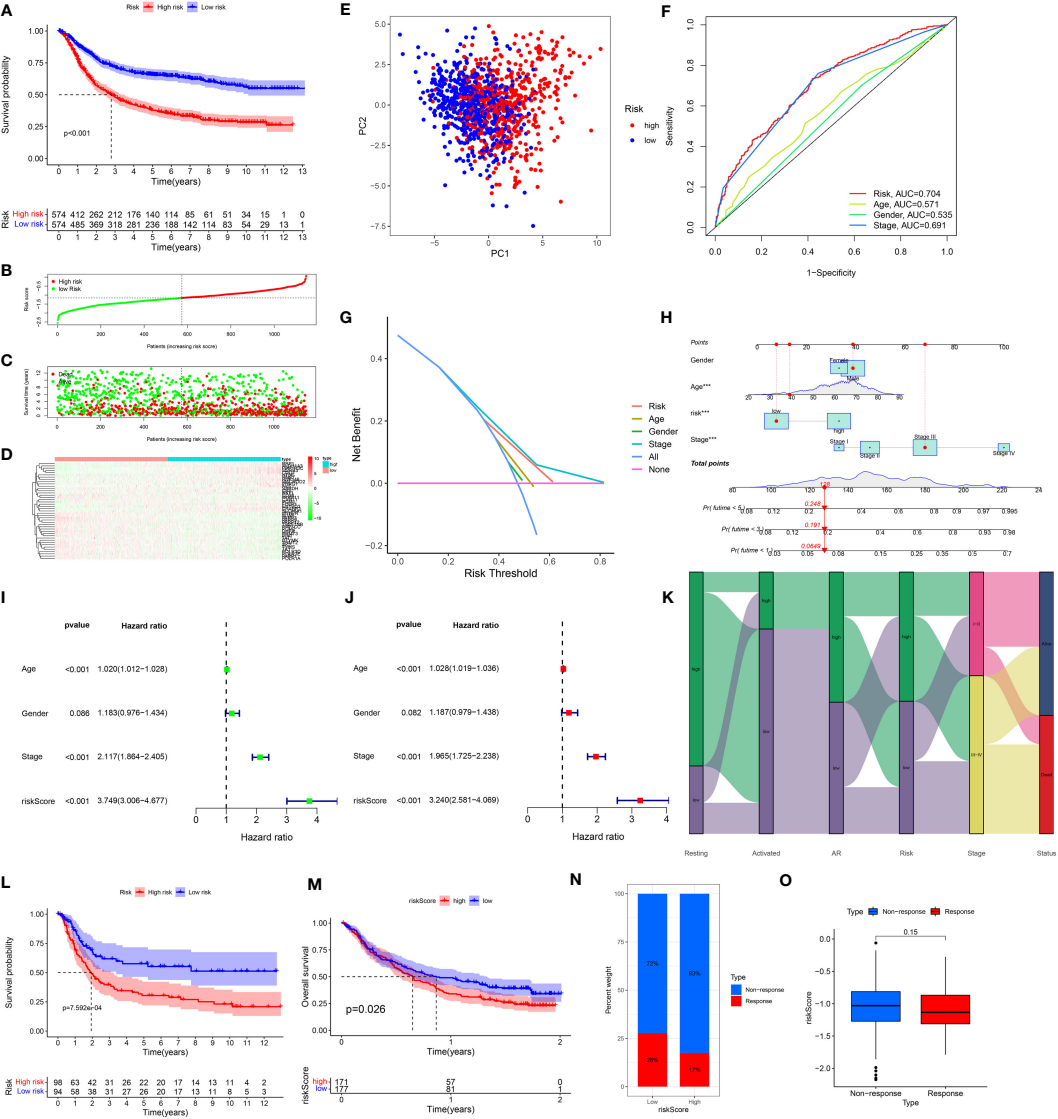
**(A)** Kaplan-Meier plots of overall survival between high- and low-risk groups in the testing group by the log-rank test. **(B–D)** The distribution of risk scores, the survival status of patients, and the expression level in screening single gene. **(E)** PCA plot of the risk scores. **(F)** ROC curve analysis of the independent prognostic factors. **(G)** DCA of the risk score. **(H)** Nomogram of GC patient OS combining the risk score and the clinicopathological variables. **(I, J)** Independent prognostic analysis of the model showed that risk score was an independent predictor. **(J)** Sankey diagram showing correlation of two CD4T cells, AR, risk score, clinical grade, and prognosis. **(K)** Risk scores were validated in the external dataset GSE15459, and low-risk OS was better than high-risk patients. **(L)** Risk scores were validated in the IMvigore210 database, and low-risk OS was better than high-risk patients. **(M)** Percentage of predicted immunotherapy responses within different risk groups in the IMvigore210 database. **(N, O)** Differences in risk scores between patients who respond and those who do not respond to immunotherapy.

To verify the predictive validity and associated characteristics of the ARMG risk model, we calculated the ARMG risk score according to the formula and divided GC patients into high- and low-risk groups in the training cohort. The results showed that patients with high risk scores had significantly worse OS than those with low risk scores (P<0.001, **Figure 4A**), with a significantly increased risk of death (**Figures 4B–D**). Using PCA analysis, we observed a distinct clustering of patients based on their risk scores (**Figure 4E**). In addition, ROC curves showed (**Figure 4F**) that the risk score might be superior to other clinical characteristics (Age, gender and stage) to predict clinical outcomes (AUC=0. 704), which was also verified by the DCA curve (**Figure 4G**). The risk scores also differed in patients with different clinical characteristics, with younger and advanced stage patients exhibited higher risk scores (**Figure S2A, C**). For clinical application, we set up a nomogram by combining risk score with age, gender, and stage (**Figure 4H**) to assess 1-, 3-, and 5-year survival for individual patients. The risk score was proved to be an independent prognostic factor (**Figures 4I, J**). Besides, the sankey diagram demonstrated the correlation between activated CD4^+^ memory T cells, resting CD4^+^ memory T cells, AR and risk score with clinical characteristics and outcomes. It showed that GC patients with lower AR levels tended to be classified in the high-risk group with advanced stage and high risk of death (**Figure 4K**).

The association of the ARMG risk score and survival outcomes was further supported with the external cohorts (GSE15459 and IMvigor210). Each patient’s risk score was calculated according to the same formula. Based on the optimum cut-off values, patients were separated into high- and low-risk groups. In both cohorts, K-M survival curves revealed a significant OS difference across the two groups. Results indicated that the high-risk group experienced significantly worse outcomes than the low-risk group (**Figures 4L, M**). The IMvigor210 cohort also revealed that the high-risk group exhibited a relatively higher proportion of non-responders to immunotherapy compared to the low-risk group (**Figure 4N**). While the risk scores in immunotherapy-responsive patients were lower than non-responders with borderline significance (**Figure 4O**).

These findings collectively suggested that risk scores computed through the risk model containing 43 genes hold a strong prognostic value and effectively stratified GC patients into high- and low-risk groups. Compared to other clinical features, this model also exhibited more reliable predictive validity in terms of monitoring patients’ survival and guiding subsequent treatment decisions.

### Predicting response to immunotherapy and chemotherapy with the ARMG risk model

**Figure 5:**
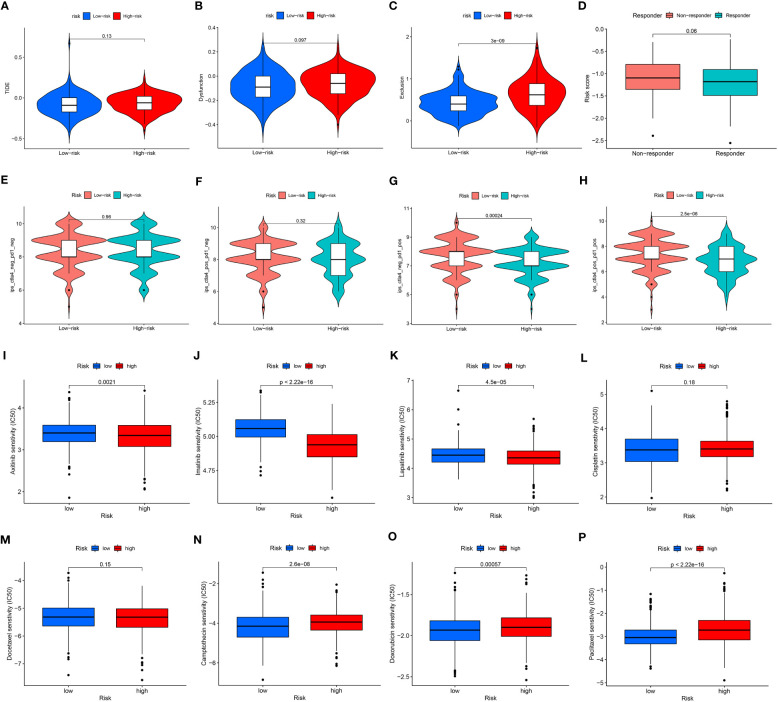
**(A)** Differences in TIDE scores between high- and low-risk groups. **(B)** Differences in immune dysfunction scores between high- and low-risk groups. **(C)** Differences in immune function rejection scores between high- and low-risk groups. **(D)** Differences in risk scores between immunotherapy responders and non-responders. **(E–H)** Difference in IPS score between high and low risk groups. (**I–P)** Differences between IC50 of chemotherapy drugs between high and low risk groups.

To fully clarify the differences between the immunotherapy responses of patients in different risk groups, we investigated the distinctions of TIDE and IPS score between the high- and low-risk groups. The TIDE score (**Figure 5A**) and the immune dysfunction score (**Figure 5B**) were at critical values between the high- and low-risk groups. But to the immune exclusion score, there was significant difference between the two groups (**Figure 5C**). In all these scores above, the high-risk group scored higher than the low-risk group. The non-responders to immunotherapy had higher ARMG risk scores than responders, which was consistent with the result predicted from IMvigor210 cohort (**Figure 5D**). It could be seen through the IPS score that there was a difference between the high- and low-risk groups in response to immune checkpoint therapy in PD-1 positive patients, regardless of CTLA4 negative or positive (**Figures 5E–H**). Similarly, the high-risk group had higher IPS scores. This reflected that the risk score model could predict not only the prognosis of GC patients, but also their immune response and guide the choice of regimen for immune checkpoint therapy.

Similarly, we also applied CGP and Cellminer databases to predict chemotherapy sensitivity between different risk groups. Axitinib, imatinib, and lapatinib had significantly higher IC50 values in the low-risk group (**Figures 5I–K**), while cisplatin and docetaxel did not differ significantly (**Figures 5L, M**). Camptothecin, doxorubicin, paclitaxel had significantly higher IC50 values in the high-risk group (**Figures 5N–P**). And the analysis of risk-chemotherapy drug correlation in the Cellminer database revealed that the effects of most chemotherapeutic drugs were negatively correlated with risk scores, while zoledronate and Irofulven were positively correlated with risk scores (**Figure S3**).

### Genomic, immune and functional landscape of GC patients with different ARMG risk scores

**Figure 6:**
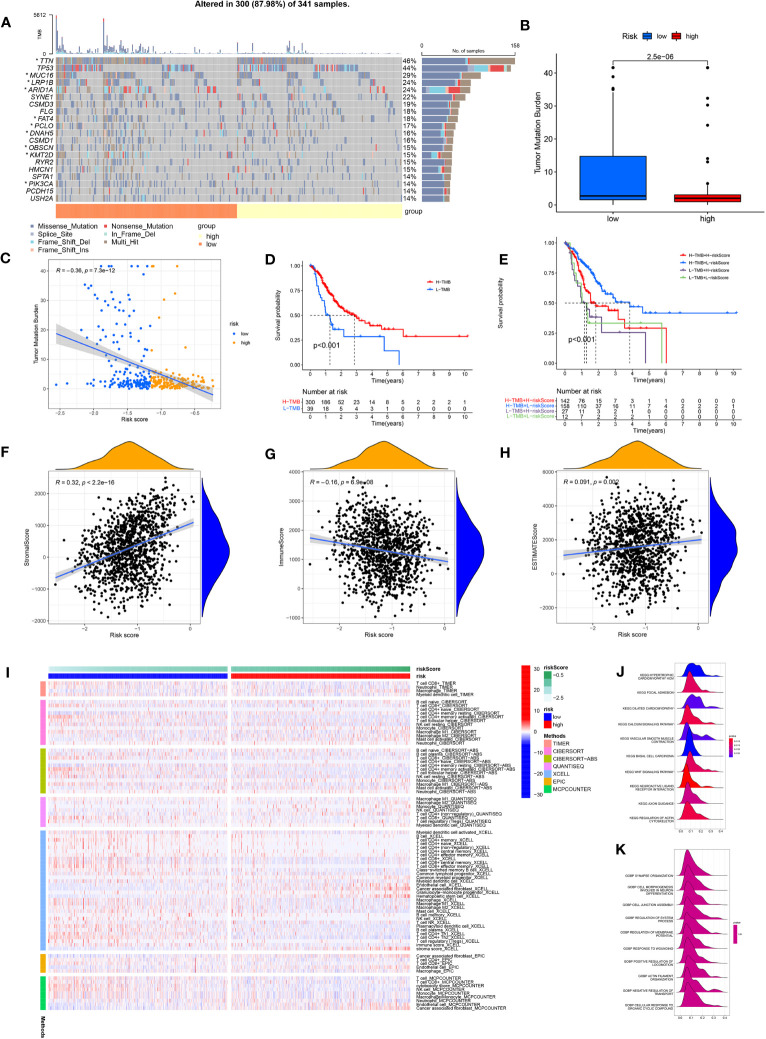
**(A)** Waterfall plot showing the TMB landscape for high- and low-risk groups. **(B)** TMB differences between high and low risk groups. **(C)** Risk score is negatively correlated with TMB. **(D)** OS differences between high- and low-TMB groups. **(E)** OS differences between the four groups (high-TMB+low-risk, high-TMB+high-risk, low-TMB+low-risk, and low-TMB+high-risk). **(F)** Risk scores were positively correlated with stroma scores. **(G)** Risk scores were negatively correlated with immune scores. **(H)** Risk scores were positively correlated with estimate scores. **(I)** Immune cell infiltration was calculated between high- and low-risk groups by seven algorithms. **(J)** KEGG analysis of the ARMG model. **(K)** GO analysis of the ARMG model.

Comparing the frequency of mutations in the high- and low-risk groups, we found that the low-risk group had a higher frequency of mutations for the majority of genes than the high-risk group did (**Figure 6A**). In addition, TMB differed significantly between the high- and low-risk groups, with the low-risk group owning a significantly higher TMB than the high-risk group (**Figure 6B**). The spearman correlation test also revealed a significantly negative correlation between TMB and risk scores (**Figure 6C**). We further investigated the possible differences in survival between patients with high and low TMB. OS considerably outperformed low TMB group in the high TMB group (**Figure 6D**). Combining TMB and risk score analysis (**Figure 6E**) revealed that patients with high TMB and low risk scores had the best OS while those with low TMB and high risk scores had the worst OS.

The model also differed in their correlation with tumor stemness, with no significant correlation with DNAss (**Figure S4A**) but a significant negative correlation with RNAss (**Figure S4B**). To investigate the variations in the immunological microenvironment between various risk categories, we found that stroma score and estimate score were both positively correlated with the risk scores, while immune score negatively correlated with the risk scores (**Figures 6F–H**). There was also a varying degree of variation in risk scores between different tumor immune subtypes (**Figure S4C**), as well as between different immune cells (**Figure S4D**) and immune functions (**Figure S4E**). We also analyzed risk scores and the 43 genes that comprise the model in relation to immune checkpoints (**Figure S4F**), m6A-related genes (**Figure S4G**), and cuproptosis-related genes (**Figure S4H**). These genes were closely associated with all of the above genes to varying degrees.

We used seven algorithms to analyze immune cell infiltration between high- and low-risk groups and found that levels of activated CD4^+^ memory T cells were higher in the low-risk group, while levels of resting CD4^+^ memory T cells was higher in the high-risk group (**Figure 6I**). GO and KEGG analyses (**Figures 6J, K**) of the main enriched pathways and functions of the model revealed that pathways were enriched in the WNT signaling pathway, basal cell carcinoma and myocardial disease signaling pathways.

### Pan-cancer analysis of ARMG risk model

**Figure 7:**
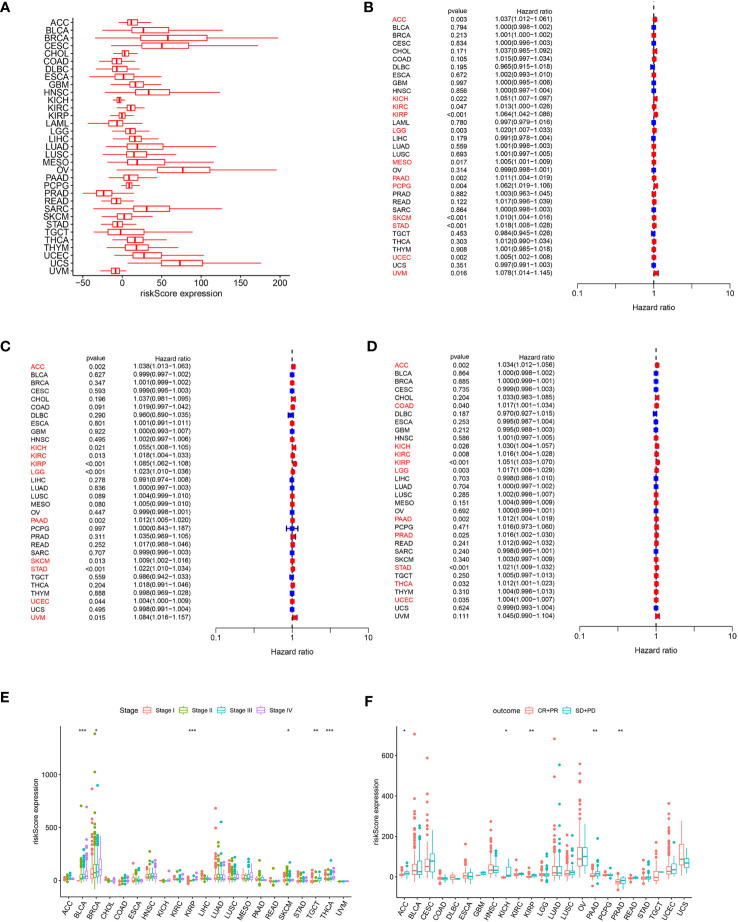
**(A)** Expression of the ARMG model in pan-cancer. **(B)** Hazard ratio of the ARMG model for OS in pan-cancer. **(C)** Hazard ratio of the ARMG model for DFS in pan-cancer. **(D)** Hazard ratio of the ARMG model for PFS in pan-cancer. **(E)** Differences in risk scores between clinical grades in pan-cancer. **(F)** Differences in risk scores between treatment outcomes in pan-cancer. *p<0.05, **p<0.01, ***p<0.001.

In order to assess the overall efficacy of the model in pan-cancer, pan-cancer cohort from TCGA was enrolled and analyzed. The levels of the model were firstly evaluated and found to be varied across different cancer types (**Figure 7A**). The model’s prognostic predictive ability was assessed for each cancer type using Cox regression analysis, specifically for OS (**Figure 7B**), DFS (**Figure 7C**), and PFS (**Figure 7D**). The risk model consistently provided predictive viability for three clinical outcomes in ACC, KICH, KIRC, KIRP, LGG, PAAD, STAD, and UCEC. Additionally, K-M curve analysis of OS showed significant differences in 20 types of cancers. Notably, the low-risk group had better OS compared to the high-risk group in most types of cancers except for DLBC, KICH, OV, and UCS (**Figure S5**).

Correlation analysis between clinical stages and risk score revealed a positive relationship in BLCA, BRCA, KIRP, SKCM, TGCT, and THCA (**Figure 7E**). Treatment response analysis (**Figure 7F**) demonstrated the predictive validity of risk score in ACC, KICH, KIRP, PAAD, and PRAD. In these cancers, higher risk scores were associated with poorer treatment response (PD+SD) compared to the group with an objective response (PR+CR). Additionally, ARMG risk scores varied depending on the patient’s gender and age in some cancer types (**Figures S6A, B**).

**Figure 8:**
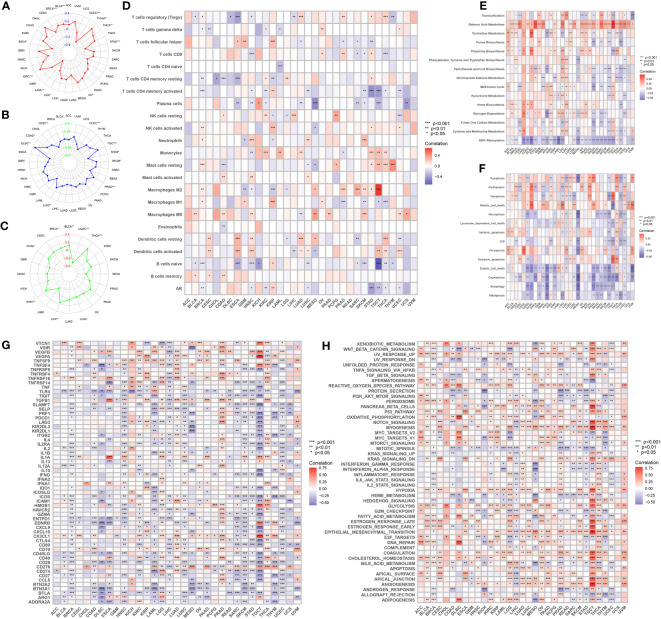
**(A)** Distribution of correlation coefficients between risk scores and TMB in pan-cancer. **(B)** Distribution of correlation coefficients between risk scores and MSI in pan-cancer. **(C)** Distribution of correlation coefficients between risk scores and neoantigen in pan-cancer. **(D)** Distribution of the correlation between immune cells, AR and risk scores in pan-cancer. **(E)** Distribution of 15 metabolism pathways correlated with risk scores in pan-cancer. **(F)** Distribution of 14 cell death-related pathways correlated with risk scores in pan-cancer. **(G)** Distribution of correlation between risk scores and immune checkpoints in pan-cancer. **(H)** Distribution of correlation between risk scores and cancer-related pathways in pan-cancer.

In addition, we analyzed the correlation of the model with TMB (**Figure 8A**), MSI (**Figure 8B**), and neoantigens (**Figure 8C**) in pan-cancer. Risk score showed the strongest negative correlation with TMB, MSI and neoantigens in UCEC, the strongest positive correlation with TMB in KIRC, with MSI-H in DLBC, and with neoantigens in THCA. Meanwhile, in STAD, both TMB and MSI-H showed significant negative correlation with risk score.

We further investigated the correlation between risk score and immune cell infiltration of each cancer type. In BRCA, KIRC, KIRP, and STAD, both resting and activated CD4^+^ memory T cells were significantly associated with risk scores. Consistent with our previous inferences, risk scores in STAD were positively correlated with resting CD4^+^ memory T cells, and negatively correlated with AR and activated CD4^+^ memory T cells. What’s interesting is that in KIRP, this phenomenon is exactly the opposite of that in STAD (**Figure 8D**).

The correlation between the risk score and metabolism pathways showed that retinoic acid metabolism is positively correlated with the model in most cancer types (**Figure 8E**). Therefore, we chose this pathway for experimental validation. Furthermore, we analyzed the correlation between the model and 14 cell death patterns (**Figure 8F**). We found that risk score was significantly associated with different types of cell death in multiple cancer types, demonstrating that risk score can influence tumor prognosis by regulating cell death. Notably, in STAD, the risk score was inversely associated with most types of cell death. The ARMG risk score showed significantly positive relation with immunoinhibitors TGFB1 and VTCN1, and was negatively related with immunostimulators CD27, CD28, CD40LG, CD80, ENTPD1 and ICOS in pan-cancer (**Figure 8G**). Correlational analysis with hallmark pathways revealed a positive association between the risk score and pro-tumorigenic pathways that promote cancer development and progression, including angiogenesis, epithelial-mesenchymal transition, glycolysis, and hypoxia pathways, as depicted in **Figure 8H**.

### Effect of all-trans retinoic acid on the transition of CD4^+^ memory T cells

**Figure 9:**
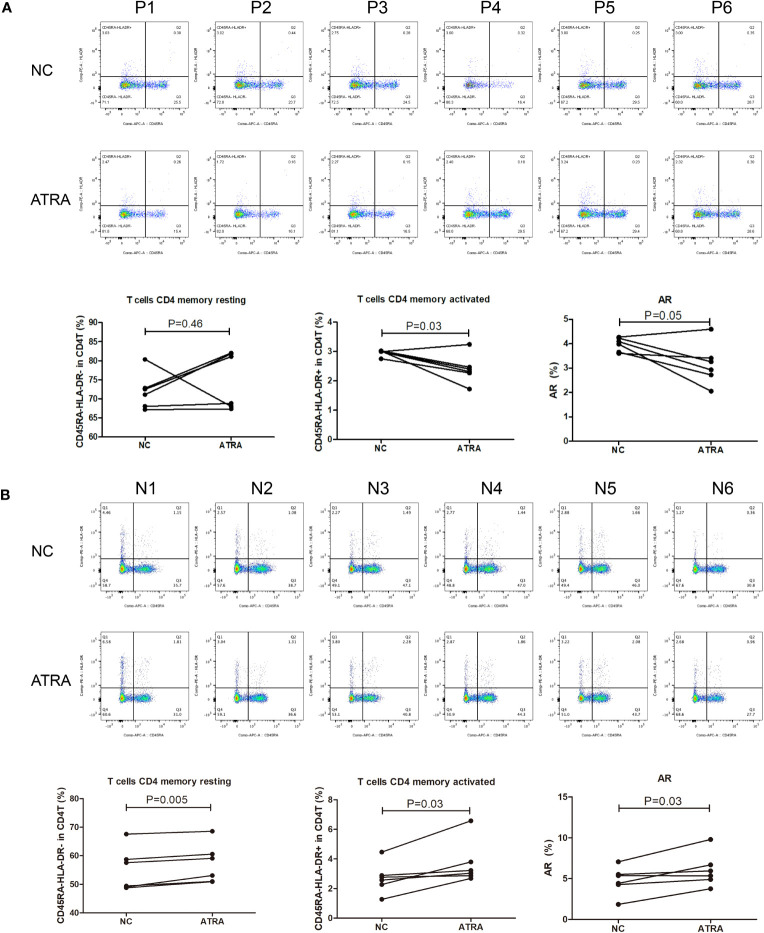
Flow cytometry for detecting the number and proportion of CD4^+^ memory T cells and AR in peripheral blood of GC patients (n=6) with advanced stages **(A)** and healthy individuals [n=6; **(B)**] after co-culture with ATRA.

Given the pervasively positive correlation with risk scores across different cancer types, we elected to investigate the effect of the retinoic acid metabolism pathway on transition of CD4^+^ memory T cells. After addition of ATRA to enhance retinoic acid metabolism, there was no statistical difference in the number of resting CD4^+^ memory T cells compared to those in NC group of GC patients. But an upward trend of resting CD4^+^ memory T cells could be assessed in five cases. On the other hand, both activated CD4^+^ memory T cells and AR were significantly inhibited in the presence of ATRA. This indicated that the conversion of resting CD4^+^ memory T cells to activated CD4^+^ memory T cells was significantly suppressed after retinoic acid metabolism pathway enhancement, thus leading to a decreased AR in GC (**Figure 9A**). However, in contrast to the GC patients, in the healthy individuals cohort, both resting and activated CD4^+^ memory T cells increased after the addition of ATRA, and the percentage of increase was dominated by the activated CD4 memory T cells, which led to an increase in AR (**Figure 9B**).

## Discussion

GC is a fatal disease with high heterogeneity in molecule and phenotype, and it has a very special tumor microenvironment, which is highly appropriate to promote tumor progression and metastasis (28, 29). Consequently, there is an imperative need to develop novel strategies to improve the survival rate of GC patients (30). Immunotherapy has emerged as a promising approach for various cancers, including GC (31). However, as our understanding of GC immunogenomics deepens, we have gained insights into the extensive heterogeneity of this disease. This highlights the necessity for effective tools to identify new predictive biomarkers and enable personalized therapy (32). Distribution of immune cell subpopulations has been shown to predict tumor behavior (33), as well as to influence treatment response (34). Therefore, we hope to find predictive biomarkers by studying the characteristics of immune cell infiltration in GC.

In the current study, we investigated the correlation between immune infiltration and prognosis of GC patients using gene expression profiles from the TCGA and GEO databases. We discovered that prognosis was significantly associated with activated CD4^+^ memory T cells: more infiltration of activated CD4^+^ memory T cells predicted better prognosis. In contrast, resting CD4^+^ memory T cells infiltration had the opposite prognostic effect, and the levels of these two cell types exhibited a negative correlation. This suggested the potential interconversion between resting and activated CD4^+^ memory T cells. The promotion of this conversion, leading to an increased AR, was linked to improved patient outcomes. The similar results could be found in the previous study which demonstrated that high abundance of CD4^+^ memory T cells was associated with better survival in GC patients (35).

CD4^+^ memory T cells are a subset of antigen-specific CD4^+^ T cells that persist after the primary T cells response’s expansion, constriction, and memory phases (36–38). It has emerged as a prognostic factor in various cancers, including kidney cancer (39), lung cancer (40), pancreatic cancer (41) and breast cancer (42, 43). Heightened memory after secondary antigen stimulation, CD4^+^ T cells multiply and develop into specialized CD4^+^ T cells subsets that are specific to pathogens (44, 45). For example, infiltration of activated CD4^+^ memory T cells was noticeably higher in colorectal cancer tissues compared to normal tissues (46). As one of hallmarks of cancer, metabolic reprogramming plays a crucial role in supporting the sustained growth of long-lived memory T cells after immune response stimulation (47). Glucose metabolism was reported to facilitate the proliferation, differentiation and function of activated T cells (48). Additionally, the activation and maintenance of homeostasis of CD4^+^ memory T cells are also dependent on glucose uptake and glycolysis (49). The involvement of glucose metabolism are also critical for the production of CD4^+^ memory T cells (50). Given these insights, we assumed that metabolic reprogramming might influence the prognosis of GC patients by affecting the transition of different states of CD4^+^ memory T cells.

We then screened 15 metabolism pathways (including the retinoic acid metabolism pathway) that were associated with both AR and prognosis to establish an ARMG risk model. We further validated the model and demonstrated that the model was independent, stable, and plausible for prognosis prediction of GC. This inference was also confirmed in the external dataset. Moreover, the model provided a means to predict the response to immunotherapy and the sensitivity to chemotherapeutic agents in GC patients, facilitating the screening of chemotherapeutic agents and determining the need for immunotherapy, which is widely used in clinical applications.

The TMB landscape of patients with different risk scores can be used for a more thoroughly clinical risk stratification of patients. Existing research has consistently shown that patients with higher TMB tend to have a more favorable prognosis (51). Our findings align with previous research, revealing that the low-risk group, characterized by lower ARMG risk scores, exhibited higher TMB. Within this low-risk group, we observed higher mutation rates in MUC16 and TTN compared to the high-risk group. Previous reports indicated that MUC16 and TTN mutations could predict high TMB level and were correlated with favorable prognosis in pan-cancer, including GC. Meanwhile, TTN mutation was associated with better response to immune checkpoint blockage in solid tumors (52, 53). Collectively, these findings provide insights into the improved prognosis observed in GC patients with low risk scores, shedding light on the potential role of TMB and specific gene mutations in patient outcomes.

Our model also owned good predictive validity in pan-cancer. However, it’s noteworthy that in a few cancer types, high risk scores were unexpectedly associated with better prognosis. This intriguing observation can be attributed to the inherent heterogeneity among different tumor types, each characterized by unique immune microenvironments and mechanisms of action. We therefore explored the different degrees of correlation between risk scores and the presence of immune cells in pan-cancer. And the 15 metabolism pathways we screened were correlated inextricably with risk scores in pan-cancer. Among them, the retinoic acid metabolism pathway was positively correlated with the model indicating that the role between this pathway and the model is consistent and stable in most cancer types. This also predicted that enhanced metabolism of retinoic acid could lead to reduced AR. Thus, to verify the role of retinoic acid metabolism pathway on AR, we conducted *in vitro* experiments using flow cytometry to detect different states of CD4^+^ memory T cells in presence of ATRA. The results were consistent with our hypothesis, as addiction of ATRA led to a decrease in both activated CD4^+^ memory T cells and AR. In the healthy individuals cohort, the addition of ATRA caused the AR to show the opposite trend, which may be due to the difference between the immune microenvironment in normal subjects and that in GC patients, which precisely indicateed that our prediction model presented specificity in GC patients. As to why this phenomenon is presented in the healthy individuals cohort and how it behaves in other cancer types, further studies are still needed.

Vitamin-like A is a retinol derivative that is necessary for epithelial differentiation and healthy embryonic development (54). ATRA, a biologically active metabolite of vitamin-like A, has shown significant promise in various malignancies, including epithelial carcinoma, precancerous lesions, and acute promyelocytic leukemia (APL) (55). The compound has been used in chemoprevention and differentiation therapy for several cancers, and has shown good efficacy in combination with pembrolizumab for metastatic melanoma (56). However, in GC, from the perspective of immune modulation, ATRA was shown to reduce the capacity of T cells to kill cancer cells by upregulating the expression of PD-L1, reducing the anticancer effects of PD-L1 antibodies, and reducing T cells activation *in vivo* (57), which is different from the effects of ATRA reported in other types of cancer (58). In our study, we further demonstrated that the retinoic acid metabolism pathway was negatively correlated with AR and that enrichment of the pathway leaded to downregulation of AR, which in turn might cause malignant progression of GC as well as poor prognosis. Though the mechanism of AR inhibition by ATRA is not yet clear, we could reasonably infer that inhibition of the retinoic acid metabolism pathway could effectively improve the prognosis of patients with advanced GC. Future research will likely focus on the development of retinoic acid inhibitors as potential therapeutic agents in GC. In addition, the use of ATRA in combination with chemotherapeutic agents or immune checkpoint inhibitors for the treatment of malignant tumors has been clinically tested in many cancer types, but the applicability of this study to GC patients may also provide a reference.

It was known that cells maintain their physiological homeostasis in a normal or stress-challenged (injury or infection, etc.) state through different cell death pathways. Both programmed and non-programmed cell death are involved in this process, suppressing or promoting tumors, partly depending on changes in the TME (59). Our study found that the model was richly associated with cell death pathways in most cancer species. In GC, most types of cell death were negatively correlated with the risk score. This demonstrated that increased AR could act as a cancer suppressor and improve prognosis by activating cell death, which might be focus for future research. In addition, the different associations between immune checkpoints and risk scores contribute to the different predictive effects of AR on prognosis in pan-cancer. Immune cells, immune checkpoints and metabolism pathways interact with each other through different pathways to have a comprehensive effect on cancer, resulting in different prognostic effects.

However, there are still some limitations in this study. Firstly, our analysis data came from public databases and lacked detailed treatment information. Secondly, multiple metabolism pathways were involved in the model, among which only retinoic acid metabolism pathway was validated with clinical samples. Further validation of other metabolism pathways and their roles in other types of cancer should be conducted in the future. Finally, the sample size of the experiment is small, and we will consider increasing the sample size and apply more experimental methods for further validation.

## Conclusions

We performed an in-depth bioinformatics analysis of the role of CD4^+^ memory T cells in GC and the mechanisms by which metabolic reprogramming affects the states of CD4^+^ memory T cells. Metabolism pathways associated with AR were screened after integrating prognostic information, and a predictive model was constructed using these associated genes. The ARMG risk model could consistently and independently predict prognosis in GC patients, with better prognosis in the low-risk group. The prognostic value of the model was validated in an external dataset. In addition, the retinoic acid metabolism pathway, which is relevant in most types of cancer, was screened for validation in the ensuing pan-cancer analysis, and these results provided new insights into GC evolution and the development of immunotherapy.

## Data availability statement

The datasets presented in this study can be found in online repositories. The names of the repository/repositories and accession number(s) can be found in the article/**Supplementary Material**.

## Ethics statement

The studies involving humans were approved by Ethics Committee of The First Affiliated Hospital of Nanjing Medical University. The studies were conducted in accordance with the local legislation and institutional requirements. The participants provided their written informed consent to participate in this study.

## Author contributions

YS: Writing – original draft. LL: Writing – original draft. YF: Writing – original draft. YL: Writing – review & editing. XG: Writing – review & editing. XX: Writing – original draft. DZ: Writing – original draft. XW: Writing – original draft. XZ: Writing – original draft.
