# Supplementary material for: Metabolic reprogramming involves in transition of activated/resting CD4+ memory T cells and prognosis of gastric cancer
Source: Front Immunol. 2023 Nov 27;14:1275461. doi: 10.3389/fimmu.2023.1275461 (PMC10711070; doi:10.3389/fimmu.2023.1275461)
Supplement: Supplementary file 1 [file DataSheet_1.docx]

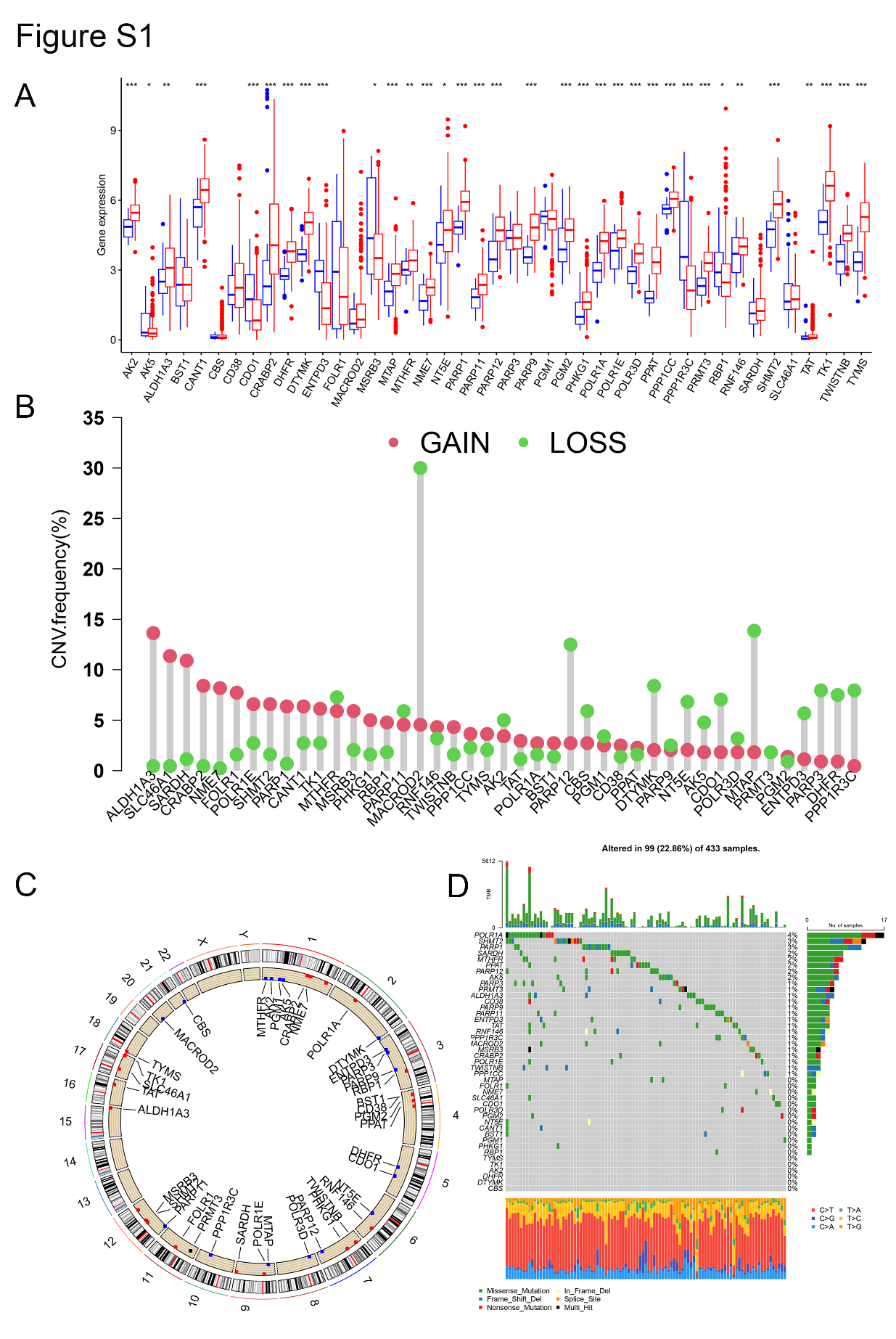


**Figure S1:** **A.** Differential expression of 43 genes for ARMG model construction between tumor tissues and normal tissues based on TCGA-STAD dataset. **B.** CNV frequencies of the 43 genes used to construct the ARMG model. **C.** The 43 genes used to construct the ARMG model were at the CNV locus on the chromosome. **D.** The TMB landscape of 43 genes used to construct the ARMG model.


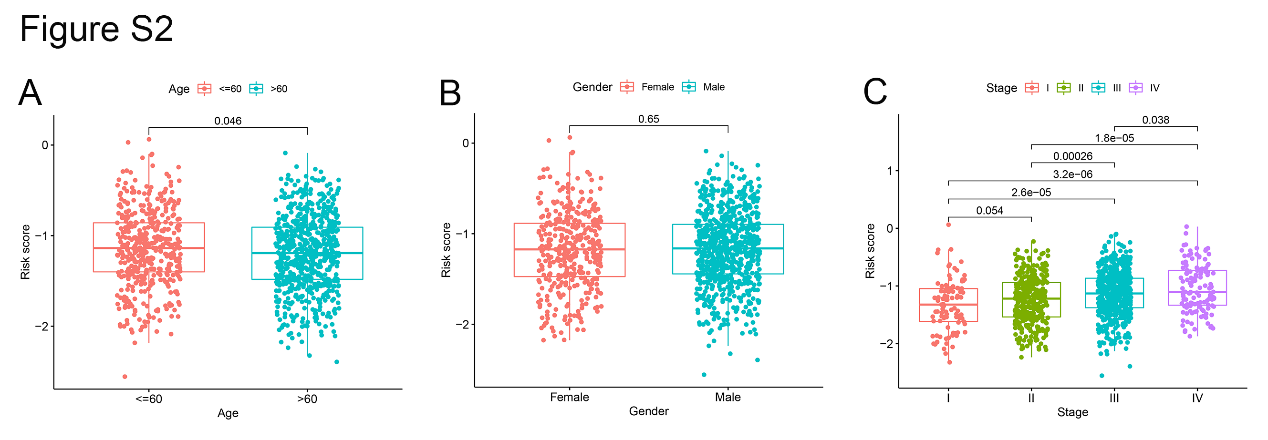
**Figure S2:** **A.** Risk scores differed between age groups (≤60 years vs. >60 years), with higher risk values in younger patients with gastric cancer. **B.** Risk scores between patients with gastric cancer by gender, showing no difference. **C.** Differences in risk scores between different clinical stages of gastric cancer patients, with higher stages associated with higher risk values.


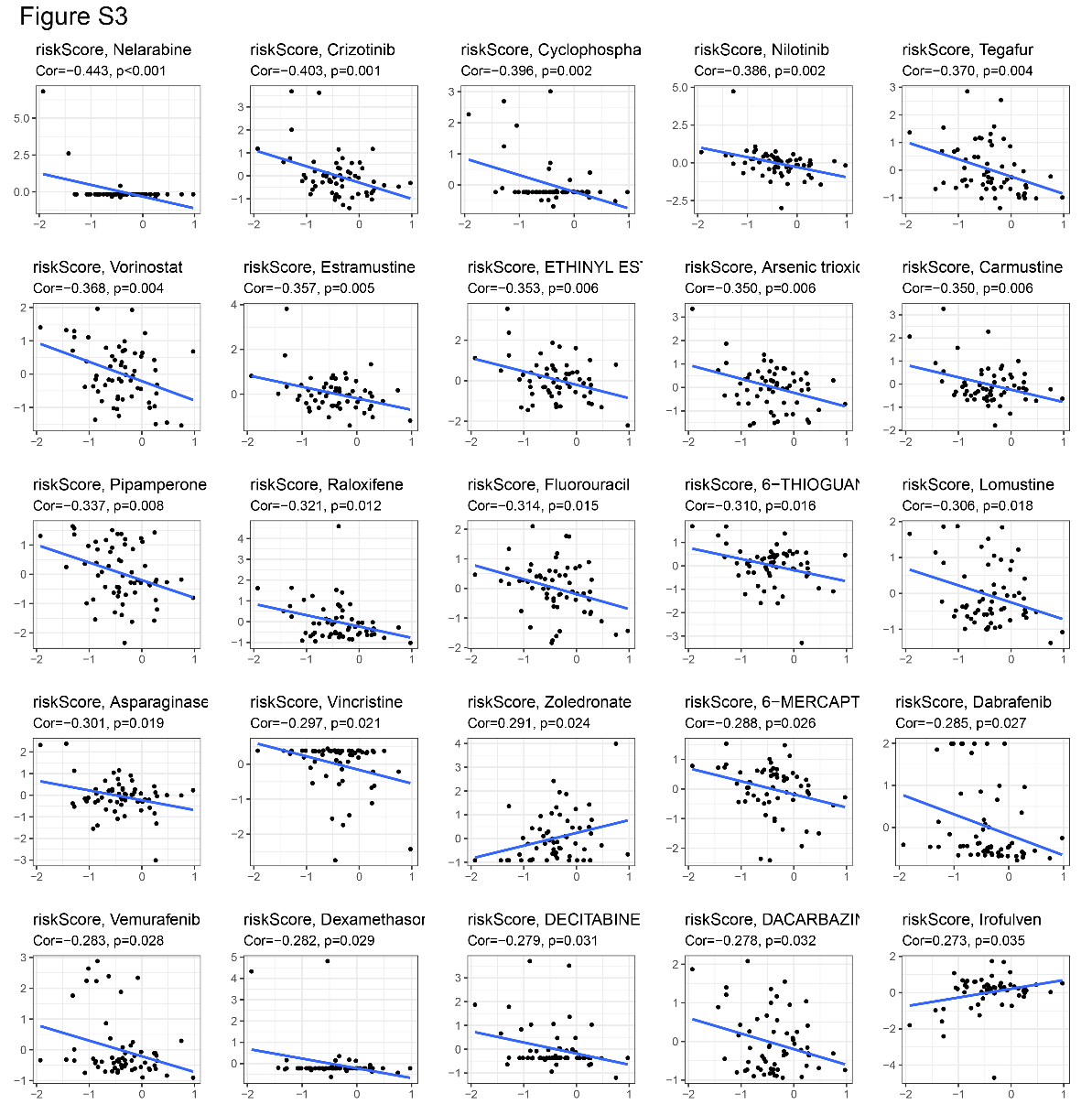


**Figure S3:** Differences in the correlation between chemotherapy drugs and risk scores in the Cellminer database.


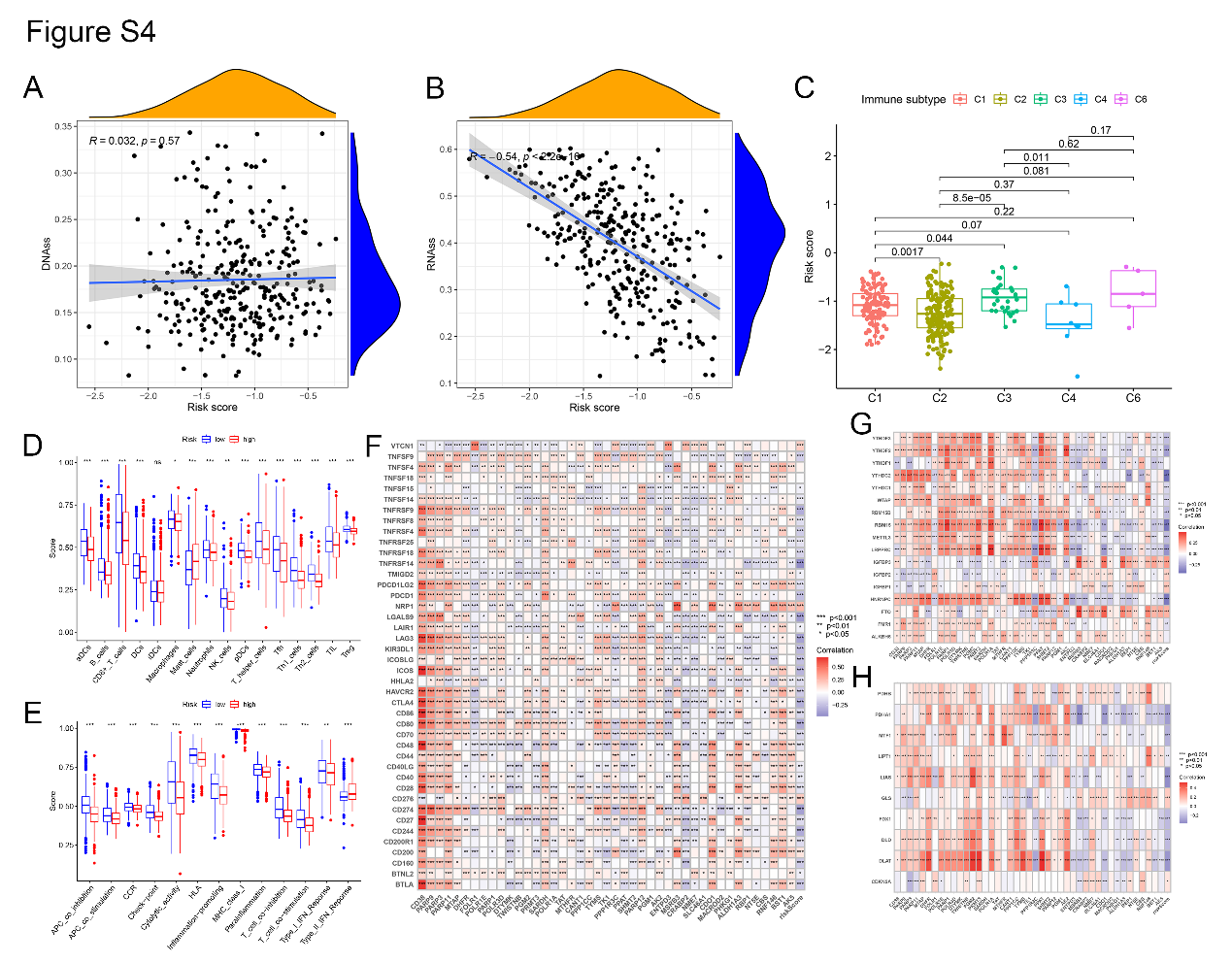


**Figure S4:** **A.** Correlation of risk scores with DNAss. **B.** Correlation of risk scores with RNAss. **C.** Differences in risk scores between different immune subtypes of gastric cancer. **D.** Differences in immune cell infiltration between high and low risk groups. **E.** Differences in immune function between high- and low-risk groups. **F.** correlation of immune checkpoints with 43 genes in the ARMG model and the risk scores. **G.** Correlation of m6A related-genes with 43 genes in the ARMG model and the risk scores. **H.** Correlation of cuproptosis related-genes with 43 genes in the ARMG model and the risk scores.


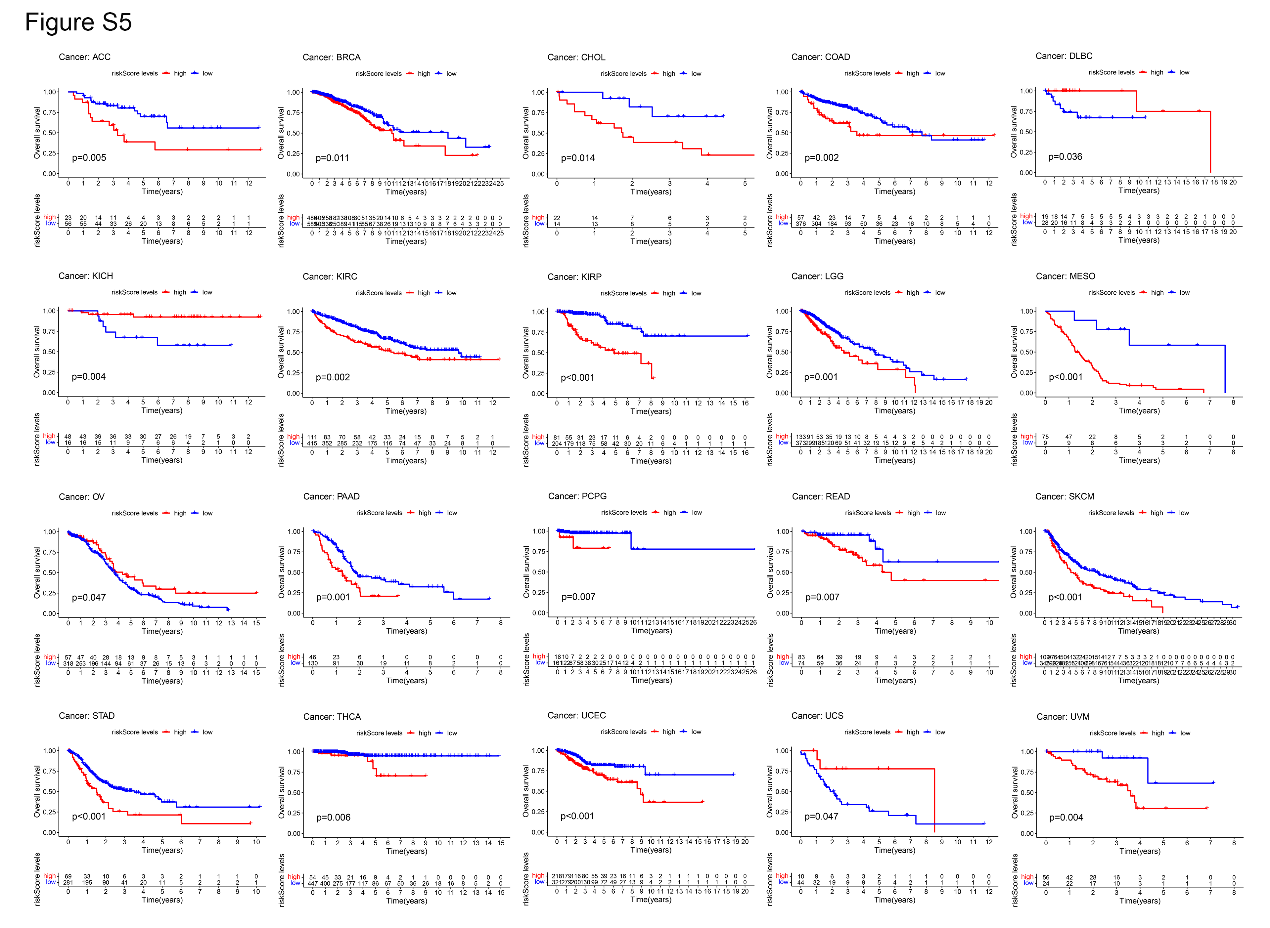


**Figure S5:** OS differences among patients with different risk scores in each cancer type were mostly consistent with gastric cancer, with a poorer prognosis for patients at higher risk.


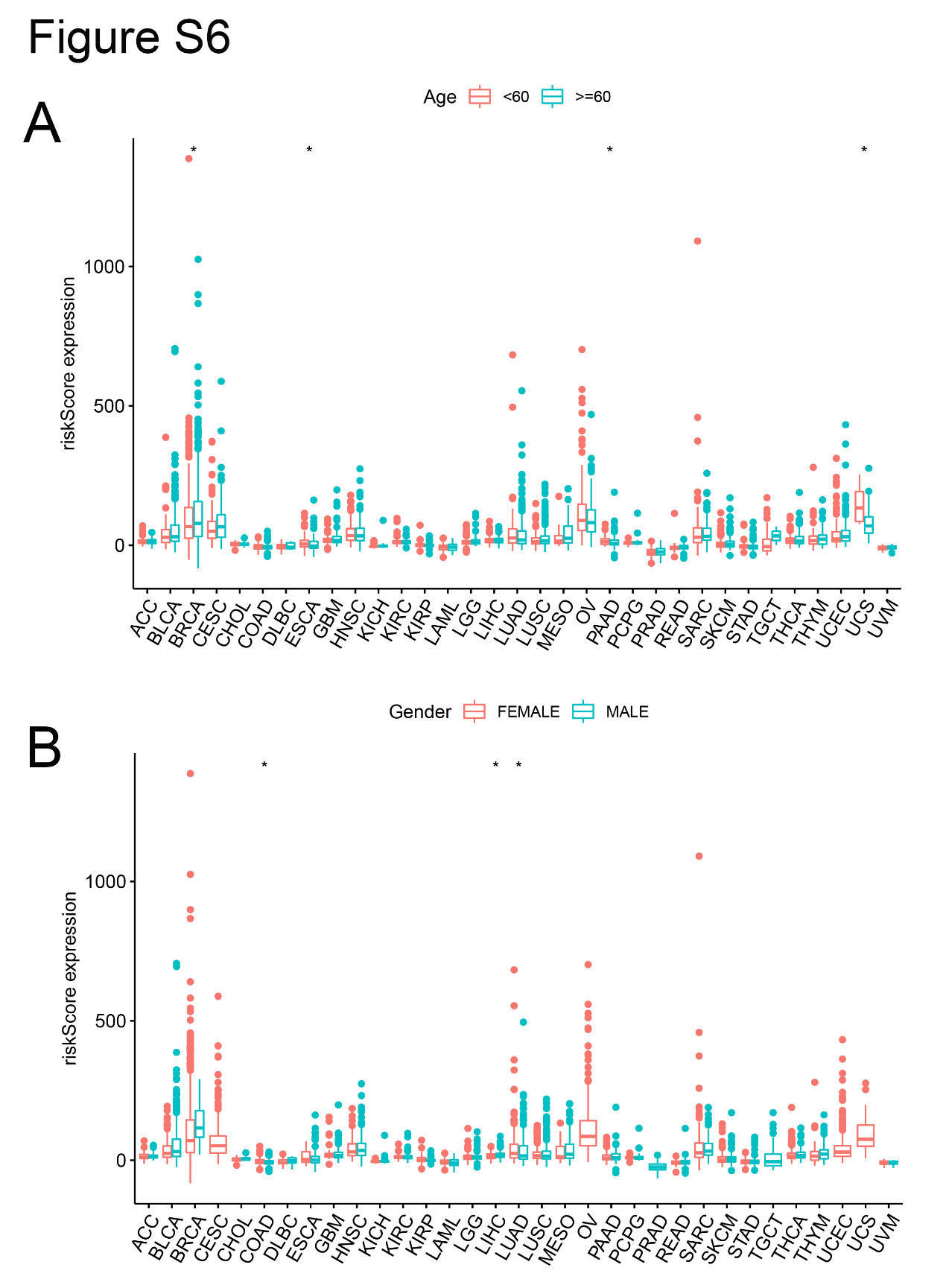


**Figure S6:** **A.** Differences in risk score expression between different age groups of patients (≤60 years vs. >60 years) for pan-cancer. **B.** Differences in risk score expression between male and female patients for pan-cancer.
